# Evaluating the effect of equine tetanus antitoxin on mortality rates of dogs affected by tetanus

**DOI:** 10.18849/ve.v9i1.676

**Published:** 2024-03-20

**Authors:** Berry Wong+

**Keywords:** ANTITOXIN, CANINE, DEATH, DOGS, MORTALITY, OUTCOME, PROGNOSIS, SEVERITY, SURVIVAL, TETANUS

## Abstract

**PICO question:**

In dogs with tetanus, does administering the equine tetanus antitoxin compared to not administering the antitoxin reduce mortality rates?

**Clinical bottom line:**

**Category of research:**

Treatment.

**Number and type of study designs reviewed:**

Three studies were reviewed for this Knowledge Summary, all of which were retrospective case-control studies.

**Strength of evidence:**

Weak.

**Outcomes reported:**

There was no difference in survival to discharge between dogs treated and dogs not treated with equine tetanus antitoxin.

**Conclusion:**

The current literature suggests that administering the equine tetanus antitoxin to dogs affected by tetanus had no positive or negative effect on mortality rates, though the level of evidence amongst the literature is weak.

## How to apply this evidence in practice

The application of evidence into practice should take into account multiple factors, not limited to: individual clinical expertise, patient’s circumstances and owners’ values, country, location or clinic where you work, the individual case in front of you, the availability of therapies and resources.

Knowledge Summaries are a resource to help reinforce or inform decision-making. They do not override the responsibility or judgement of the practitioner to do what is best for the animal in their care.

## Clinical scenario

A 10-month-old female neutered Golden Retriever is presented to you with a 2 day history of having a ‘strange’ facial expression and generalised muscle stiffness. Upon presentation, the dog is exhibiting *risus sardonicus*, characteristic of tetanus. The use of the equine tetanus antitoxin has been described in the management of tetanus, but is there any evidence to suggest any benefits between dogs that do receive the equine tetanus antitoxin compared to those that do not?

## The evidence

A search of the literature revealed three studies relevant to this PICO, all of which were retrospective case-control studies. The strength of evidence for each paper is considered weak due to the lack of prospective systematic reviews or meta-analyses for this Knowledge Summary.

## Summary of the evidence

**Bandt et al. (2007):** 

Population	Dogs with characteristic clinical signs and treated for tetanus, without evidence of other neuromuscular disease.
Sample Size	20 dogs.
Intervention Details	A total of 16 dogs received the equine tetanus antitoxin (200 IU/kg, intravenously [IV]), 12 of which also received an intradermal test dose of 0.1 ml prior to the IV dose. Amongst the 16 dogs, six dogs were found to have wounds or lacerations and received an additional 1000 IU equine tetanus antitoxin after exploration and lavage. A total of four dogs did not receive the antitoxin. All dogs were treated with penicillin G (30,000 IU/kg, IV, q8hr). Eight dogs were treated with metronidazole (10 mg/kg, IV, q8hr). All dogs received muscle relaxants and sedatives as necessary including diazepam (0.5 mg/kg, IV, q4–6hr), acepromazine (0.05–0.07 mg/kg, IV, q3–4hr), and methocarbamol (55–100 mg/kg, IV, q30–60 mins). All dogs received IV fluid and nutritional support.
Study design	Retrospective case-control study.
Outcome studied	Survival – this was defined as survival to discharge. Each case was described as ‘survived’, ‘died’ or ‘euthanised’. Severity – there was no mention as to how this was specifically measured, though the following was recorded and presumed to be taken into account with respect to assessing severity: clinical signs and complications measured as number of cases affected respectively; and duration of hospitalisation for survivors, first sign of improvement of clinical signs, and time to complete recovery, all of which were measured as a median value and a range respectively. Duration – this was defined as time to complete recovery amongst dogs that survived their respective treatment protocol, and measured as a median value and a range.
Main findings (relevant to PICO question)	There was no difference in survival to discharge between dogs treated and dogs not treated with antitoxin. Survival – 50% (2/4) dogs that did not receive the antitoxin survived, and 50% (8/16) dogs that received the antitoxin survived. The researchers also reported no difference in severity or duration of clinical signs between dogs treated and not treated with antitoxin. This is difficult to verify given the lack of clarity with respect to specific measurement of severity, and specific comparison of time to complete recovery for dogs treated with antitoxin and those that were not.
Limitations	No power calculation was used, nor was there any form of randomisation. Small sample size. Variable treatment protocols in each case as well as advances in nursing care over the 16 year duration of the study make it difficult to extrapolate reliable conclusions. The study mentions a Chi-square analysis was used but no such data was presented. Information bias associated with retrospective nature.

**Burkitt et al. (2007):** 

Population	Dogs with typical clinical signs of tetanus, excluding those with incomplete medical records, hypocalcaemia, and confirmed neurotoxicoses or myositis. A canine tetanus severity classification system was developed prior to identifying dogs eligible for inclusion into the study. The system was based on the human classification system, information gathered from veterinary textbooks and case reports, and the researchers’ experience. The dogs were then grouped into the following classes: Class I – facial signs of tetanus only. Class II – generalised rigidity or dysphagia, with or without class I signs. Class III – class I or II signs that were recumbent or had seizures. Class IV – class I, II, or III signs, with abnormal heart rate, respiratory rate, or blood pressure measurements.
Sample Size	38 dogs.
Intervention Details	29 dogs were treated with the tetanus antitoxin, 28 of which received a median dose of 326.5 IU/kg with a range of 10–1900 IU/kg, intravenously (IV). Eight dogs were not treated with the tetanus antitoxin. Information on the use of tetanus antitoxin was not available for one dog. 21 dogs were treated with metronidazole, two dogs with penicillin, and 10 dogs with both antimicrobials. 26 dogs, all of which were within classes II, III, or IV, were treated with varying sedative drugs, including acepromazine, chlorpromazine, diazepam, midazolam, pentobarbital, phenobarbital, baclofen, methocarbamol, morphine, and butorphanol. 29 dogs were nutritionally supported.
Study design	Retrospective case-control study.
Outcome studied	Day of antitoxin administration – this was measured as a median value and a range, with day 0 being the first day of illness. Progression of clinical signs – this was defined by worsening of classes within the tetanus severity classification system developed. Mortality – this was measured as survival to day 28, with day 0 being the first day of illness.
Main findings (relevant to PICO question)	The researchers reported no association between earlier antitoxin administration and progression of clinical signs or 28 day mortality rate, but there was no obvious direct comparison between those that received the antitoxin and those that did not, with respect to the day of antitoxin administration, progression of clinical signs, and mortality. Day of antitoxin administration – median of day 3 with a range of day 0 to day 13. Progression of clinical signs – 64 % (23/36) dogs developed more severe clinical signs after first evaluation in terms of progression to higher classes. One dog did not have details of first evaluation recorded, and one dog had no abnormal findings on first physical examination and therefore was not initially classified with the canine tetanus severity classification system. Mortality – 77 % (27/35) dogs survived to day 28. Three dogs were not included in the survival analyses as they either died from reasons unrelated to tetanus or were lost to follow-up.
Limitations	No power calculation was used, nor was there any form of randomisation. Small sample size. *C. Tetani* is difficult to culture, so definitive diagnosis is hard to achieve clinically. Diagnostic testing was not performed to completely rule out other neurotoxicoses or generalised myopathies. Variable intensive care management between dogs in the population given the retrospective nature of the study.

**Zitzl et al. (2022):** 

Population	Dogs with characteristic signs of local or generalised tetanus at presentation, excluding those with incomplete medical data, a history or suspicion of neurotoxic substance ingestion, ionised hypocalcaemia of < 0.8 mmol/L on admission, and findings consistent with myositis, meningoencephalitis, or spinal trauma. Dogs were classified according to a class scheme in terms of assessing disease severity: Class I – facial signs of tetanus only. Class II – generalised rigidity or dysphagia, with or without class I signs. Class III – class I or II signs that were recumbent or had seizures. Class IV – class I, II, or III signs, with abnormal heart rate, respiratory rate, or blood pressure measurements.
Sample Size	42 dogs.
Intervention Details	24 dogs received the tetanus antitoxin, one of which received tetanus antiserum of human origin, and the remaining receiving equine tetanus antitoxin. The equine tetanus antitoxin dose administered was known in 18 dogs and this was a median dose of 357 IU/kg with a range of 86–1666 IU/kg. Time from onset of tetanic signs to antitoxin administration was available for 17 dogs and this was a median of 97 hr with a range of 25–149 hr. 18 dogs did not receive the tetanus antitoxin. Information on mortality was not available for two of the 18 dogs that did not receive the tetanus antitoxin. 40 dogs were treated with antibiotics. 36 dogs received sedatives. 22 dogs received methocarbamol, 33 dogs were given magnesium, and atropine was administered to 18 dogs. 24 dogs were supported nutritionally, 17 of which had a percutaneous endoscopic gastrotomy tube placed, four of which received an oesophagostomy tube, and three of which received a nasogastric tube.
Study design	Retrospective case-control study.
Outcome studied	Treatment outcome – this was measured as survivors or non-survivors, with survivors being defined as survival to discharge in inpatients and uneventful recovery in outpatients, and non-survivors being defined as death related to tetanus, either spontaneous or by euthanasia.
Main findings (relevant to PICO question)	Antitoxin use was not significantly different between survivors and non-survivors. Treatment outcome – 75 % (18/24) dogs that received the antitoxin were survivors, and 75 % (12/16) dogs that did not receive the antitoxin were survivors, but the p-value was 1 and so this was deemed statistically insignificant. Conversely 25 % (6/24) dogs that received the antitoxin were non-survivors, and 25 % (4/16) dogs that did not receive the antitoxin were non-survivors. Non-survivors who received the antitoxin earlier at a median time of 50 hr (26–51 hr) compared to a median time of 99 hr. (25–149 hr) in the disease course were associated with a poorer prognosis.
Limitations	No power calculation was used, nor was there any form of randomisation. Small sample size. Non-standardised treatment and timings of treatment, as well as monitoring measures used given the study’s retrospective nature. Euthanasia for non-medical reasons, e.g. financial limitations, would have affected the results. Univariable analyses as opposed to multivariable analyses were used.

## Appraisal, application and reflection

Three studies were reviewed for this Knowledge Summary, all of which were retrospective case-control studies that aimed to evaluate the clinical courses and outcomes of dogs affected by tetanus. The main finding reported was that there was no positive or negative effect on the survival of dogs affected by tetanus who received the equine tetanus antitoxin (Bandt et al., 2007; Burkitt et al., 2007; and Zitzl et al., 2022).

There were an insufficient number of cases in the studies reviewed, as demonstrated by the relatively small sample sizes. Two of the studies appraised are relatively old being published at least 15 years ago, and therefore advances in intensive care management and general nursing care since would play a part in affecting the mortality rate of dogs affected by tetanus, questioning the applicability of these studies’ findings. Furthermore, referral populations were assessed and therefore findings may not necessarily represent those found with other subpopulations. Variable treatment protocols for each case associated with the retrospective nature of the studies is a large confounding factor but this was acknowledged in their respective discussions. Given the broad aim of the studies, direct comparison of outcomes on the basis of clinical treatment choice, or specifically on the basis of administration of the antitoxin in this case, was not always clear to avoid confounding by indication as mentioned in Burkitt et al. (2007). In other words, an accurate and reliable association between the use of the equine tetanus antitoxin and mortality rates cannot be deduced based on these retrospective studies owing to the fact that more severely affected dogs were potentially more likely to receive earlier, higher frequency of treatments and interventions. As a result, alternative associations between the multiple different treatments and interventions used, as well as the indications of their use and the mortality rates of the dogs affected by tetanus, cannot be ruled out.

Cases of tetanus in dogs are relatively uncommon. The small sample sizes of the studies reviewed may be explained by the fact that dogs are relatively resistant to tetanus due to poor tetanospasmin penetration of neural tissue compared to that in humans (Greene, 2006). Moreover, the antitoxin acts by binding to any unbound toxin. Considering this, its administration would likely be useful during the peracute stage of the disease which may well be prior to presentation. Future large, randomised, prospective studies assessing for optimal timing and dosing of equine tetanus antitoxin administration, complication rates, and duration of hospitalisation or time to recovery in survivors are indicated to determine whether the findings of these three studies reviewed are supported or refuted.

## Methodology

**Search strategy:** 

Search terms	CAB Abstracts on OVID Platform (1973 to 2023 Week 39)Medline on OVID Platform (1946 to October 2023)
Dates searches performed:	CAB Abstracts: exp dogs/ (dog* or canine* or canid* or bitch*).mp. [mp=abstract, title, original title, broad terms, heading words, identifiers, cabicodes] 1 or 2 tetanus/ or clostridium tetani/ or neonatal tetanus/ (tetanus or clostridial or clostridium or tetani or clostridium tetani or c tetani or lockjaw).mp. [mp=abstract, title, original title, broad terms, heading words, identifiers, cabicodes] 4 or 5 3 and 6 (tetanus antitoxin* or TAT).mp. [mp=abstract, title, original title, broad terms, heading words, identifiers, cabicodes] (mortality or mortalities or mortality rate* or survival or survival rate* or death or dead).mp. [mp=abstract, title, original title, broad terms, heading words, identifiers, cabicodes] 7 and 8 and 9 Medline: Dogs/ (dog* or canine* or canid* or bitch*).mp. [mp=title, book title, abstract, original title, name of substance word, subject heading word, floating sub-heading word, keyword heading word, organism supplementary concept word, protocol supplementary concept word, rare disease supplementary concept word, unique identifier, synonyms] 1 or 2 Tetanus/ (tetanus or clostridial or clostridium or tetani or clostridium tetani or c tetani or lockjaw).mp. [mp=title, book title, abstract, original title, name of substance word, subject heading word, floating sub-heading word, keyword heading word, organism supplementary concept word, protocol supplementary concept word, rare disease supplementary concept word, unique identifier, synonyms] 4 or 5 Tetanus Antitoxin/ (tetanus antitoxin* or TAT).mp. [mp=title, book title, abstract, original title, name of substance word, subject heading word, floating sub-heading word, keyword heading word, organism supplementary concept word, protocol supplementary concept word, rare disease supplementary concept word, unique identifier, synonyms] 7 or 8 (mortality or mortalities or mortality rate* or survival or survival rate* or death or dead).mp. [mp=title, book title, abstract, original title, name of substance word, subject heading word, floating sub-heading word, keyword heading word, organism supplementary concept word, protocol supplementary concept word, rare disease supplementary concept word, unique identifier, synonyms] 3 and 6 and 9 and 10
Search terms	06 Oct 2023

**Exclusion / inclusion criteria:** 

Exclusion	Full text not available in the English language. Lack of relevance to the PICO question. Lack of online full text availability. Conference proceedings, abstracts, literature reviews, single case reports, case series, book chapters, opinions, and letters.
Inclusion	All appropriate articles relevant to the PICO.

**Table 1:** 

Database	Number of results	Excluded – Not available in the English language	Excluded – Irrelevant to the PICO question	Excluded – Conference proceedings, abstracts, literature reviews, case reports, case series, book chapters, opinions, and letters	Total relevant papers
CAB Abstracts	4	0	2	1	1
Medline	4	0	2	0	2
Acquired paper					1
Total relevant papers when duplicates removed					2

## ORCiD

Berry Wong: https://orcid.org/0000-0003-3619-4049

## Conflict of interest

The authors declare no conflicts of interest.
